# Cell type specific polyploidization in the royal fat body of termite queens

**DOI:** 10.1186/s40851-023-00217-6

**Published:** 2023-10-11

**Authors:** Tomonari Nozaki, Eisuke Tasaki, Kenji Matsuura

**Affiliations:** 1 Laboratory of Insect Ecology, Graduate School of Agriculture, Kyoto University, Kyoto, 606-8502 Japan; 2 Laboratory of Evolutionary Genomics, National Institute for Basic Biology, Okazaki, 444‐8585 Japan; 3 Department of Biology, Faculty of Science, Niigata University, 8050 Ikarashi 2-No-Cho, Nishi-Ku, Niigata, 950-2181 Japan

**Keywords:** Somatic polyploidy, Social insects, Caste differentiation, Isoptera, Queen fecundity, Fat body, Cell type, Flow cytometry

## Abstract

Tissue-specific endopolyploidy is widespread among plants and animals and its role in organ development and function has long been investigated. In insects, the fat body cells of sexually mature females produce substantial amounts of egg yolk precursor proteins (vitellogenins) and exhibit high polyploid levels, which is considered crucial for boosting egg production. Termites are social insects with a reproductive division of labor, and the fat bodies of mature termite queens exhibit higher ploidy levels than those of other females. The fat bodies of mature termite queens are known to be histologically and cytologically specialized in protein synthesis. However, the relationship between such modifications and polyploidization remains unknown. In this study, we investigated the relationship among cell type, queen maturation, and ploidy levels in the fat body of the termite *Reticulitermes speratus*. We first confirmed that the termite fat body consists of two types of cells, that is, adipocytes, metabolically active cells, and urocytes, urate-storing cells. Our ploidy analysis using flow cytometry has shown that the fat bodies of actively reproducing queens had more polyploid cells than those of newly emerged and pre-reproductive queens, regardless of the queen phenotype (adult or neotenic type). Using image-based analysis, we found that not urocytes, but adipocytes became polyploid during queen differentiation and subsequent sexual maturation. These results suggest that polyploidization in the termite queen fat body is associated with sexual maturation and is regulated in a cell type-specific manner. Our study findings have provided novel insights into the development of insect fat bodies and provide a basis for future studies to understand the functional importance of polyploidy in the fat bodies of termite queens.

**Supplementary Information:**

The online version contains supplementary material available at 10.1186/s40851-023-00217-6.

## Background

Polyploid cells are widespread among various tissues and organs that exhibit high metabolic activity in plants and animals. They play pivotal roles in the regulation of gene expression, cell size, and differentiation [1–4]. Polyploid cells are generated by endocycles, or DNA amplification during the S phase of the cell cycle without subsequent cell division [2, 5]. Endocycles have been considered a low-cost strategy to increase cell and/or tissue size and to efficiently produce massive amounts of molecules needed during development, reproduction, immunity, and other life activities. This is because this process can avoid spending more time, materials, and energy to undergo normal mitosis [2, 5, 6]. However, there is still insufficient data connecting ploidy levels with cell specialization, gene expression levels/patterns, and organ development, which is required to determine the adaptive functions of tissue-specific polyploidy [6, 7].

The insect fat body is a multifunctional organ that is involved in the synthesis, storage, and secretion of lipids, proteins, and carbohydrates [8]. During oogenesis, vitellogenins or yolk protein precursors are massively produced in the female fat body and accumulate in developing oocytes [8, 9]. Sexually mature females of some solitary insects have more polyploid cells in their fat bodies than in other tissues or organs, which are most likely to boost vitellogenin production [10–12]. In fact, it has been demonstrated that in the locust *Locusta migratoria*, inhibition of polyploidization in the female fat body resulted in suppression of oogenesis [13], although the detailed cytological mechanism remained unclear. The insect fat body generally contains several cell types: adipocytes (or trophocytes), oenocytes, urocytes and bacteriocytes [8, 14, 15]; therefore, cytological information together with ploidy dynamics would provide further implications about the mechanisms and functional roles of fat body polyploidy.

**Fig. 1:**
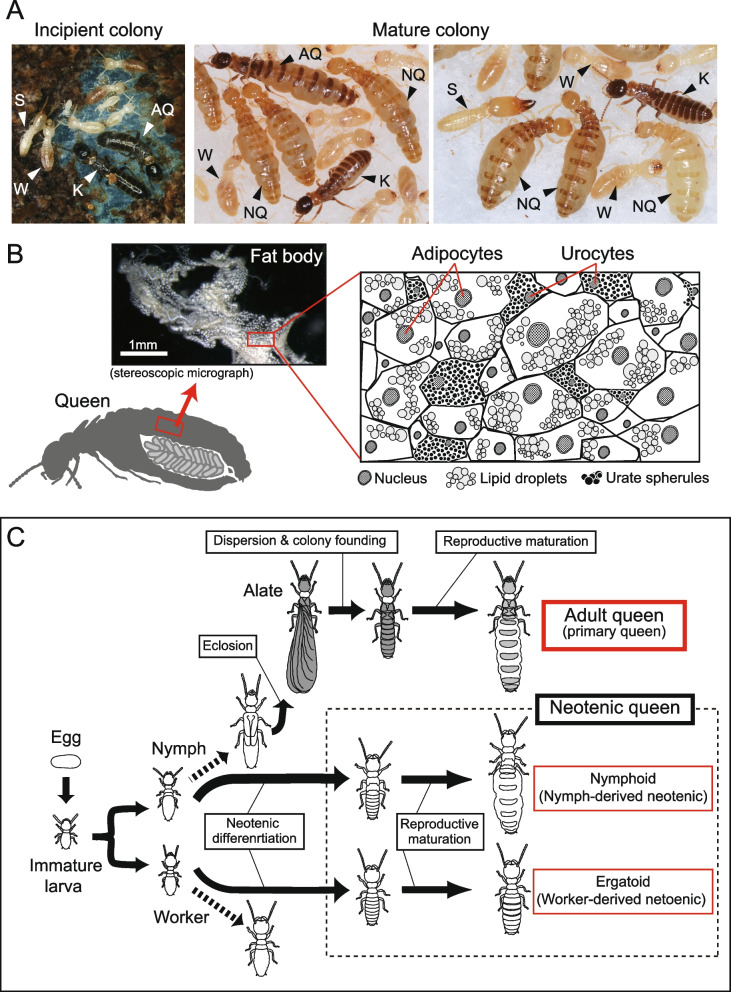
Queen development and the fat body structure in termites. **A** Photographs of *Reticulitermes speratus* colonies. Monogamous pairs of adults found new colonies. Mature field colonies contain a number of queens, which continuously produce eggs over several years. AQ, adult queen; K, king; NQ, neotenic queen; S, soldier; W, worker. **B** The abdomen of a fully matured termite queen is almost filled with ovaries and fat body. In *R. speratus*, the fat body appears as whitish loose tissue located around the digestive tube and reproductive organs. The fat body tissue is comprised of two types of cells, that is, adipocytes, which are metabolically active and contain lipid droplets, and urocytes, which are not active but contain urate spherules. The illustration of the histology of the termite fat body was drawn with reference to [14]. **C** Developmental patterns of queens in *R. speratus*. Queens are categorized into adult queens (primary queens), nymphoids (nymph-derived neotenics), and ergatoids (worker-derived neotenics). All queens undergo reproductive maturation after adult or neotenic molts. In *R. speratus*, almost all queens collected in field colonies are nymphoid queens. Ergatoid queens are rarely observed in field colonies [27], yet they can easily be induced under laboratory conditions [28]

In social insects, which are characterized by a reproductive division of labor (Fig. 1A) [16], an intriguing relationship between tissue-specific polyploidy and the division of labor has been identified [17–21]. Nozaki and Matsuura [20, 21] demonstrated that in termites, high endopolyploidy only occurs in the queen fat body, but not in males or non-reproductive females. They also reported that highly fecund queens in foraging termite species had higher polyploid levels in their fat bodies than queens in wood-dwelling species, which were less fecund. Recently, Séité et al. [22] conducted ploidy analysis on the fat body of a higher termite, *Macrotermes natalensis*, in addition to transcriptomics, lipidomics, and metabolomics analyses. They showed that the ploidy level of the fat body of adult queens increased with the development of reproductive traits, and that this polyploidization occurred rapidly after colony founding and egg laying initiation [22]. These studies hypothesized that fat body polyploidy enhances egg production and is linked to histological and/or cytological modifications during reproductive maturation [14, 23–26], while there are few studies focusing on both the polyploidization and cytological changes in the fat body.

The termite fat body is composed of two types of cells, namely, urocytes and adipocytes (Fig. 1B) [8, 14]. Urocytes are packed with urate spherules, which deposit uric acid as a nitrogen reservoir. Adipocytes are metabolically active cells that synthesize and store lipids, glycogen, and various proteins including vitellogenins. Only the primitive termite *Mastotermes darwiniensis* has a third cell type, that is, bacteriocytes, that harbor the bacterial symbiont, which is ubiquitous in cockroaches [14, 15]. The fat bodies of functional queens contain more adipocytes than urocytes, and the adipocytes of fully mature queens specialize in the synthesis of proteins [14]. In higher termites, the fat body of queens contains abundant proteins and RNAs, which have shown considerable differences from those of nonreproductives (royal fat body; [23, 24]). Besides, a recent study clearly demonstrated transcriptional downregulation of genes related to energy storage, and upregulated expression of genes involved in protein synthesis in the royal fat body [22]. Based on these observations, adipocytes are likely to show higher ploidy levels than urocytes. However, to date, there has been no information on the cellular specificity of fat body polyploidization.

*Reticulitermes* is a well-studied termite genus in terms of its reproductive biology, oogenesis or ovarian development, and caste differentiation (Fig. 1C) [27, 29–36], and the complete genome has recently been provided [37]. In *R. speratus*, most mature field colonies contain tens to hundreds of neotenic queens [27, 34, 35], which are usually derived from nymphs [27, 36]. Meanwhile, nymph-derived and worker-derived neotenics can be induced under laboratory conditions [28, 36, 38]. Adult queens (or primary queens) that were derived from imagos are occasionally collected from field colonies [27], and colony initiation using collected alates under laboratory conditions is frequently conducted by many researchers (Fig. 1A) [33, 39, 40]. Therefore, this species is suitable for comparative studies of queen types and their maturation levels.

In this study, we investigated the relationship between cell type and ploidy level in the fat body of the termite *R. speratus*. Specifically, we examined whether adipocytes and/or urocytes became polyploid during queen maturation. We first conducted microscopic observations of the cytological features of the termite fat body and confirmed the presence of two types of cells, that is, adipocytes, which contain many lipid droplets, and urocytes, which have many spherical crystals surrounding the nucleus. It was also confirmed that vitellogenin genes were highly expressed in the fat body of queens using quantitative RT-PCR. Using flow cytometry, we examined the ploidy dynamics in the fat bodies of three types of termite queens in this species, that is, nymph- or worker-derived neotenics and adult queens. Finally, our image-based analysis of nuclear size and ploidy levels showed cell specificity in the polyploidization of termite queen fat bodies: the highly doubling cell population was adipocytes. Based on our results, we have discussed the functional and developmental importance of fat body polyploidization during queen maturation and fecundity.

## Methods

### Microscopic observation of adipocytes and urocytes in the termite fat body

To visualize the differences between adipocytes containing lipid droplets and urocytes with accumulation of urate crystals, we performed a morphological analysis of the fresh fat body of *R. speratus* using a confocal laser-scanning microscope (FV1000, Olympus). The fat bodies of workers and late-instar nymphs, collected from pine or cedar forests in Aichi, April 2021, were dissected in phosphate-buffered saline (PBS; 33 mM KH_2_PO_4_, 33 mM Na_2_HPO_4_, pH 6.8). This process was conducted with fine forceps under a stereomicroscope (Olympus SZX7, Olympus). The fat body of the termites appeared as whitish loose tissue located around the digestive tube and reproductive organs (Fig. 1B) [14]. We dissected the fat body from the abdomen of the insects with care to avoid contamination by other tissues, such as Malpighian tubules and tracheoles. The dissected fat bodies were treated with trypsin buffer (0.25% trypsin [Nacalai Tesque] in PBS) for 15 min to loosen the adhesion between the cells. The tissues were stained using Hoechst 33,342 (1 μg/mL, Dojindo) and Lipi-Red (1 nmol/mL, Dojindo) in PBS for 15 min. The tissues were directly mounted on a glass slide, crushed using a cover slip, and visualized using fluorescent and differential interference contrast (DIC) microscopy. Note that we first tried to observe fat bodies fixed with 4% paraformaldehyde (PFA) in PBS, permeabilized by 0.3% Triton X-100 in PBS, and stained with 4,6-diamidino-2-phenylindole (DAPI) (1 μg/mL; Dojindo, Japan). However, this was not suitable because no urate crystals were observed, potentially because of the dissolution of urates during sample processing. We also conducted histological observations of the fat bodies of workers and queens collected in August 2018 from a field colony in Kyoto. The dissected fat bodies were fixed overnight in PBS with 2% glutaraldehyde and 4% PFA at 4 °C and postfixed with 1% OsO_4_ for 2 h. The samples were then dehydrated and embedded in epoxy resin. Ultrathin sections were stained with 2% saturated uranyl acetate solution and 2.5% lead citrate solution and examined using transmission electron microscopy (Hitachi-7650, Hitachi). The electron microscopy study was supported by the Division of Electron Microscopy, Center for Anatomical Studies, Graduate School of Medicine, Kyoto University.

### Ploidy analysis of the fat body of termites using flow cytometry

**Fig. 2:**
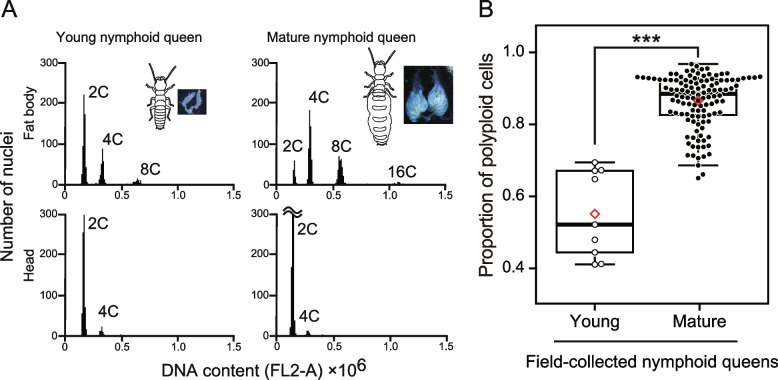
Ploidy analysis from flow cytometry of the fat body and head cells from young and matured queens from field colonies. **A** Examples of the histograms obtained for young and matured queens are presented. In each histogram, the first peak corresponds to the distribution of 2C-DNA nuclei, whereas the second, the third, and the fourth correspond to the distribution of 4C- 8C- and 16C-DNA nuclei, respectively. The ploidy was determined by analysis of the head cells (diploid; 2C, [19]). Images of insects and ovaries were included. **B** The proportion of polyploid cells in the fat body between young and mature field-collected nymphoid queens. Box plots show the median (center line), 75th percentiles (top of box), 25th percentiles (bottom of box), and whiskers connect the largest and smallest values within 1.5 interquartile ranges. The red diamonds indicate mean percentages of each category. Dots are individual values. Asterisks indicate a significant difference (linear mixed-effect model with a Wald chi‐square test, ***p < 0.05)

To investigate the ploidy dynamics in the fat bodies of queens in *R. speratus*, we compared the ploidy levels of fat bodies among queens at different maturation stages. This analysis was conducted for all three queen types in the species, that is, nymph-derived neotenic (nymphoid), worker-derived neotenic (ergatoid), and adult queen (Fig. 1C). Details of the three types of termite queen are presented in the Supplementary Information (SI Methods and Table S1). The fresh body weights of individual termites were measured using a digital balance and expressed in milligrams to two decimals. Fat bodies were dissected in PBS using fine forceps under a stereomicroscope (Olympus SZX7; Olympus). We used the heads of all of the individuals analyzed as diploid tissues [20, 21]. Tissues were processed for flow cytometric analysis using the Cycletest PLUS DNA Reagent Kit (Becton Dickinson). All of the procedures were performed according to [20, 21]. Nuclei were stained using propidium iodide (PI) and analyzed for DNA-PI fluorescence with an Accuri C6 Flow Cytometer (Becton Dickinson) at an excitation wavelength of 488 nm. Approximately 1,000 cells were acquired for each measurement. Gating was performed using Accuri C6 software v1.0.264.21 to measure the number of nuclei at each ploidy level (2C, 4C, and 8C) per sample. Representative histograms are shown in Fig. 2.

The proportion of polyploid cells in the fat body, which was calculated by the nuclei count with value of 2C as diploid, and 4C, 8C, and higher as polyploid, was compared among different maturation stages of nymphoid, ergatoid, and adult queens, and the ones from which they were derived, namely nymphs, workers, and alates. We used the worker data presented in [21]. The proportion of polyploid cells in the fat body of each individual was analyzed using generalized linear mixed-effect models (GLMMs) with binomial errors and a logit-link function. In this analysis, the category of individuals (e.g., nymphs, nymphoid 0 days, and reproducing nymphoid queens in “nymphoid queens”), and body weight were treated as fixed effects. Original colony was included as a random effect. The significance of the fixed effects was assessed by type-II ANOVA using Wald chi-square tests. Then the Tukey’s HSD post hoc test was used for comparison of the proportion of polyploid cells among the three groups (i.e., the category of individuals). All analyses were conducted using the “car,” “emmeans,” “lawstat,” “lme4”, and “multcomp” packages in R software v4.1.1 (https://www.r-project.org/). Additionally, “beeswarm” was used to draw the beeswarm plots. Differences were considered significant when the *p* value was < 0.05.

### Analysis of ploidy, cell types, and the size of nuclei in the fat body using an image-based method

To investigate the relationship between cell type and ploidy level, cell cycle analysis by flow cytometry was not suitable, because nuclei were isolated and purified from the cytoplasm, which is the key for identification of cell types. Moreover, it was difficult to surgically separate urocytes from adipocytes due to their distribution (Fig. 1B). Therefore, we established an image-based method in which the nuclear size was used as a proxy of the ploidy level. We measured and compared the nuclear size between two types of cells in the fat body of termites.

First, we confirmed the relationship between the ploidy level and size of nuclei using a cell sorting technique and microscopic observation. Nuclei sorted from the head and fat body of queens, according to each ploidy level (2C, 4C, and 8C), were measured (see SI Methods). Then, using semi-destructive treatment and microscopy, we compared the nuclear size, as a proxy for ploidy (see Results), between two types of cells, adipocytes and urocytes, in the fat bodies of workers, nymphs, and young and mature nymphoid queens. The fat bodies of workers and nymphs from two field-collected colonies (HM210907A and OZ210902) and young and reproducing nymphoid queens from lab-orphaned colonies derived from the colonies were dissected in PBS (for details see Table S1). For each colony, one individual of each termite type was randomly selected (n = 2 for each type). The tissue was treated with trypsin buffer (0.25% trypsin in PBS) for 10 min to loosen the adhesion between cells. The tissues were stained with Hoechst 33,342 in PBS (1 μg/mL, Dojindo) for 10 min. The stained tissues were directly mounted on a glass slide, crushed using a cover slip, and visualized using fluorescent and differential interference contrast (DIC) microscopy. Nuclei were randomly captured using a DS-Fi1 CCD camera (Nikon, Japan). The cell type and their number were recorded based on the DIC images. The size of the nuclei was measured using Hoechst fluorescent images, with the image analysis software ImageJ (National Institutes of Health, USA, http://rsb.info.nih.gov/ij/).

The size of nuclei from workers, nymphs, young, and reproducing nymphoid queens was compared between adipocytes and urocytes. In this analysis, we used linear mixed-effect models (LMMs), wherein the cell type and category of individuals were fixed effects, and the original colony was a random effect. The significance of the fixed effects was evaluated with type-II ANOVA using Wald chi-square tests, and Tukey’s HSD post-hoc test was used for comparison of the size of nuclei. All analyses were conducted using the “car,” “emmeans,” “lawstat,” “lme4,” and “multcomp” packages in R software v4.1.1. Additionally, “beeswarm” and “vioplot” were used to draw the beeswarm and violin plots, respectively. Differences were considered significant when the *p* value was < 0.05.

## Results

### Morphological observation of the termite fat body

Consistent with previous observations in other termites [14, 23, 24], the fat body of *R. speratus* consisted of cells that contained numerous lipid droplets and urate spherules, corresponding to adipocytes and urocytes, respectively (Fig. S1). Confocal microscopy showed that the nuclei of adipocytes were circular and varied in size, whereas those of urocytes were slightly deformed and small (Fig. S1A). The TEM observations were consistent with those of previous reports on other termite species. Two types of cells were recognizable, that is, cells with lipid droplets or urate crystals (Fig. S1B). Based on these observations, we cytologically confirmed the presence of termite adipocytes and urocytes (Fig. S1C).

### Ploidy dynamics in the termite queen fat body

#### Nymphoid queens

**Fig. 3:**
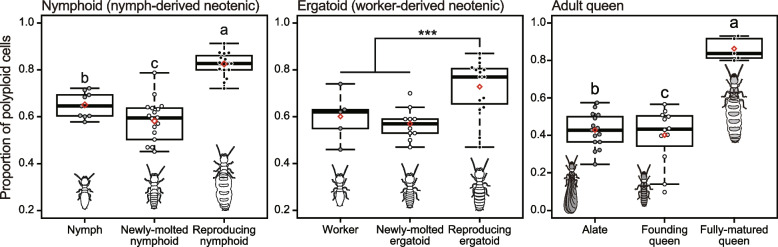
The proportion of polyploid cells in the fat body along with development of three types of queens. Box plots show the median (center line), 75th percentiles (top of box), and 25th percentiles (bottom of box), and whiskers connect the largest and smallest values within 1.5 interquartile ranges. The red diamonds indicate mean percentages of each category. Dots are individual values. Different letters and asterisks indicate significant differences among means in each comparison group (Tukey’s HSD test, *p* < 0.05)

Among the field-collected nymphoid queens, we recognized “young” and “matured” queens, as shown in the Methods. While diploid cells were dominant in the fat bodies of young queens, tetraploids (i.e., 4C cells) were always the most abundant ploidy class in the fat bodies of mature queens (Fig. 2A). The fat body of mature nymphoid queens contained significantly more polyploid (2C, 4C, 8C, and 16C) cells than that of the young queens (GLMM with type II Wald chi-square test, types of queens: *χ*^2^ = 1280.49, *df* = 1, *p* < 0.01, Fig. 2B). There were also significant effects of fresh body weight on the proportion of polyploid cells in fat bodies (body weight: *χ*^2^ = 154.93, *df* = 1, *p* < 0.01). Among the female nymphs and laboratory-induced nymphoid queens, there were significant differences in the proportion of polyploid cells in the fat body (GLMM with type II Wald chi-square test, female category: *χ*^2^ = 139.0634, *df* = 2, *p* < 0.01; Fig. 3). Body weight did not significantly affect the degree of polyploidy (body weight: *χ*^2^ = 0.0101; *df* = 1; *p* = 0.92). Reproducing nymphoids exhibited the highest proportion of polyploid cells in their fat bodies, and newly molted nymphoids showed significantly lower values than nymphs (Tukey’s HSD, *p* < 0.01, Fig. 3). In all individuals, the head cells predominantly consisted of diploid cells.

#### Ergatoid queens

Among the female workers, newly molted ergatoids, and reproducing ergatoid queens, there were significant differences in the proportion of polyploid cells in the fat body (GLMM with type II Wald chi-square test, female category: *χ*^2^ = 18.918, *df* = 2, *p* < 0.01; Fig. 3), considering the significant effect of body weight (body weight: *χ*^2^ = 35.265, *df* = 1, *p* < 0.01). Reproducing ergatoid queens had a higher proportion of polyploid cells in their fat bodies than newly molted ergatoids and female workers (Tukey’s HSD, *p* < 0.01, Fig. 3). In all individuals, the head cells predominantly consisted of diploid cells.

#### Adult queens

There were originally four categories for this type of queen, that is, female alate, field-collected founding queen, founding queen in laboratory-established colonies, and field-collected fully matured queens. However, we pooled field-collected founding queen and ones in laboratory-established colonies as “founding queens” (for details see SI Results). Among the female alates, founding queens, and fully mature queens, there were significant differences in the proportion of polyploid cells in the fat body (GLMM with type II Wald chi-square test, female category: *χ*^2^ = 226.84, *df* = 2, *p* < 0.01; Fig. 3), considering the significant effect of body weight (body weight: *χ*^2^ = 196.29, *df* = 1, *p* < 0.01). Fully matured queens had the highest proportion of polyploid cells in their fat bodies, with founding queens showing significantly lower values than the female alates (Tukey’s HSD, *p* < 0.01, Fig. 3). In all individuals, the head cells predominantly consisted of diploid cells.

### Cell-type-specific polyploidy in the fat body of termite queens

We assessed the relationship between nuclear size (area) and ploidy level. There was a significant difference in size among 2C nuclei sorted from the head sample and 2C, 4C, and 8C nuclei sorted from fat body samples (Fig. S6; GLMM with type II Wald chi-square test, nuclear category: *χ*^2^ = 431.94, *df* = 3, *p* < 0.01). Although there were some overlaps, nuclei with higher ploidy levels exhibited significantly larger sizes (Tukey’s HSD: 2C from head = 2C < 4C < 8C from fat body, *p* < 0.01; Fig. S6). This clear correlation allowed us to use nuclear size as a proxy for the ploidy level.

**Fig. 4:**
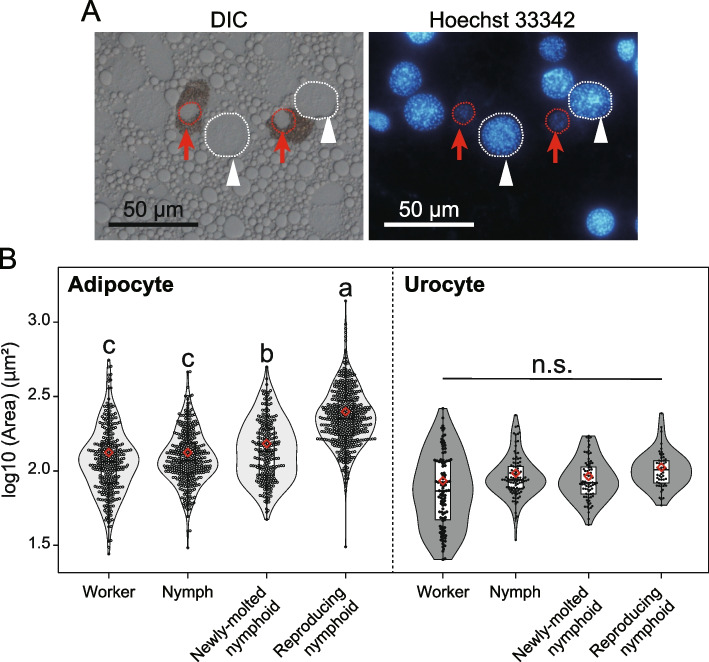
The relationship between cell types and nuclear size (a proxy of ploidy level) in the fat body of *R. speratus*. **A** Under DIC, adipocytes can be discriminated from urocytes, because adipocytes were not surrounded by urate spherules. Nuclei of both types of cells were stained and visualized by Hoechst 33,342. White arrowheads indicate representative nuclei of adipocytes (enclosed by white dashed lines). Red arrows indicate urocyte nuclei (enclosed by red dashed lines). **B** Size distribution of nuclei of adipocytes and urocytes in the fat body of worker, nymph, and immature and mature nymphoid queens. Violin plots showing the distribution of the size (area) of nuclei. Box plots show the median (center line), 75th percentiles (top of box), and 25th percentiles (bottom of box), and whiskers connect the largest and smallest values within 1.5 interquartile ranges. The red diamonds indicate mean size of each category. Dots are individual values. Different letters indicate significant differences among means in each comparison group (Tukey’s HSD test, *p* < 0.05)

Based on DIC microscopy, we confirmed that we could morphologically distinguish between the two cell types. Nuclei surrounded by urate spherules and lipid droplets were categorized into urocytes and adipocytes, respectively (Fig. 4A). There were significant differences in the proportions of adipocytes and urocytes. Reproducing nymphoids had more adipocytes (89.8%) than workers (76.7%), nymphs (78.8%), or newly molted nymphoids (76.1%) (Fisher’s exact test with Bonferroni correction, *p* < 0.001). We then investigated the relationship between cell type and nuclear size and found that both cell type and individual type had significant effects on nuclear size (LMM with type II Wald chi-square test, cell type: *χ*^2^ = 156.357, *df* = 1,* p* < 0.01; individual type: *χ*^2^ = 501.615, *df* = 3, *p* < 0.01). This interaction was significant (*χ*^2^ = 58.853, *df* = 3, *p* < 0.01). The size of adipocytes significantly increased with queen differentiation and maturation (Tukey’s HSD; worker = nymph < newly molted nymphoid < reproducing nymphoid, *p* < 0.01, Fig. 4B). Meanwhile, there was no significant difference in the size of urocytes among workers, nymphs, and queens (Tukey’s HSD, *p* < 0.01, Fig. 4B). These findings indicated that the polyploid cell population was adipocytes rather than urocytes because nuclear size can be used for a proxy for ploidy level. Note that in both cell types, the size variation was relatively large, regardless of termite phenotype and developmental stage, as suggested in the analysis of the sorted nuclei (Fig. S6).

## Discussion

In the present study, we have demonstrated that during the maturation of termite queens, their fat bodies become polyploid in a cell type-specific manner. Our flow cytometric analysis of field-collected (Fig. 2B) and laboratory-grown (Fig. 3) queens showed that the ploidy levels of the fat bodies were higher in reproducing queens that had fully developed ovaries than in young or newly molted ones, as recently shown in a higher termite [22]. This pattern was maintained regardless of the queen phenotype, that is, nymph-derived neotenic, worker-derived neotenic, and adult queens (Fig. 3). We also found that adipocytes increased their ploidy levels in the fat bodies of termite queens (Fig. 4B). In adipocytes, nuclear size, which is a proxy for ploidy levels (Fig. S5) increased significantly during queen maturation. In contrast, the size of urocyte nuclei was not significantly different between female workers and young/mature queens (Fig. 4B). The queen fat body was confirmed to highly express conventional vitellogenin genes (SI Results, Fig. S2), and “adipocyte” was assumed to be a principal cell type in metabolic activity of the fat body [8, 14]. Therefore, these results were compatible with the idea that the polyploidization in the fat body could contribute to increasing egg production or massive vitellogenin synthesis [21]. In future studies, gene expression, cell types, and ploidy levels should be examined in detail, alongside performing cell sorting techniques and single-cell-level gene expression analysis.

The dynamics of the ploidy level and the proportion of cell types that we described in the present study are consistent with previous histological studies on the development of “royal fat body” [23, 24]. The fat bodies of functional queens in higher termites contain a larger number of adipocytes than urocytes, and the adipocytes of fully matured queens are specialized in the synthesis of proteins. They have limited lipid content but abundant rough endoplasmic reticulum and Golgi apparatus [14]. These observations were supported by molecular level analyses by Séité et al. [22]; in fact, the fat body of mature queens had low level of triglycerides, but exhibited abundant diglycerides, which are lipids utilized in oogenesis [22]. Upregulation of the expression levels of genes involved in the synthesis of proteins was also demonstrated in that work [22]. Specialization of adipocytes through polyploidization may be essential for the formation of the “royal fat body” in termite queens. The relationship between royal fat body development and polyploidy should be addressed using integrated approaches, including cytological, histological, and omics analyses.

There have been several reports on age-related polyploidy in insect tissues and organs [17, 41]. However, this study has shown that increasing the ploidy level in the fat body of termite queens is closely linked to reproductive maturation rather than being age-related. This is because 3 months after emergence from artificially established colonies, queens exhibited almost the same degree of ploidy in their fat bodies as field-collected queens that would otherwise have been bred for several years (unknown exact age) (Figs. 2B and 3). Data from a higher termite also suggested that ploidy levels in the fat bodies of queens increased sharply during the early stages of reproductive maturation [22]. Therefore, rather than aging promoting fat body polyploidy per se (the possibility cannot be ruled out), fat body polyploidy is likely to play an essential role in high reproductive outputs and the extraordinarily long lives of termite queens, such as increasing egg production or prevention of aging [42, 43].

Juvenile hormone (JH) promotes fat body cell polyploidization in locusts [10, 11, 13, 44, 45]. In termites, JH plays critical roles in neotenic and soldier differentiation [46–48]. In general, insect JH is important for ovarian maturation and oogenesis [49]. Therefore, polyploidy in the fat body of neotenic queens is likely the result of neotenic differentiation with increasing JH titers. Nevertheless, we found that polyploidization of the termite fat body occurred during queen maturation rather than during neotenic differentiation (Fig. 3). The fat bodies of newly molted neotenics (nymphoids [58%] and ergatoids [57%)] had a similar proportion of polyploid cells as nymphs (65%) and workers (60%). However, these proportions were significantly different from those of reproducing neotenics, including nymphoids (82%) and ergatoids (73%) (Fig. 3). Alates (42%) and founding (young) adult queens (40%) had a lower proportion of polyploid cells in their fat bodies than fully mature adults (86%) (Fig. 3). After initiation of the colony, female alates (founding queens) oviposit a small number of eggs for a month [40, 50]. This indicated that high-level polyploidy in the fat body is not essential for oogenesis, as suggested in [22]. Fat body polyploidization may be critical for the mass production of eggs, and/or long-term active reproduction by termite queens, although this should be assessed more directly in future studies.

Our results from flow cytometry indicated slight but significant decreases in the rate of polyploid cells in the fat body during queen differentiation and maturation (nymphoid and adult queens, Fig. 3). Also, in ergatoid queens, the level of polyploidy decreased in the newly molted neotenics, although it was not statistically significant. Cell division much faster than endoreduplication may increase the population of daughter (diploid) cells and cause a decrease in the ratio of polyploid cells. Therefore, these drops of polyploid ratio imply the tissue remodeling, including proliferation, death, and differentiation of cells, in the fat bodies during neotenic/adult molts and subsequent queen maturation. Investigation of the post-embryonic development and tissue remodeling in the termite fat body would deepen our understanding of the formation process of the royal “polyploid” fat body of termite queens.

In conclusion, our results have demonstrated that not urocytes, but adipocytes, become polyploid in the fat body of termite queens during reproductive maturation. To our knowledge, this is the first report that shows cell-type specificity in the polyploidization of the insect fat body. Our data also emphasized that fat body polyploidy was associated with queen maturation, that is, “the formation of the royal fat body,” rather than being from aging or caste differentiation. Therefore, it is necessary to investigate whether endopolyploidy plays a role in increasing or regulating the transcription of rRNA and mRNA per nucleus, as shown in plants [7]. Our study has provided a foundation for further molecular and single-cell level analyses of the functional roles and proximate mechanisms of polyploidy in insect fat bodies.

### Supplementary Information

**Additional file 1:** **SI Methods.** **SI Results.** **Table S1.** Sample information of R. speratus colonies used for ploidy analysis. **Figure S1.** Cell types in the termite fat body. **Figure S2.** Vitellogenin gene expression in the termite queen fat body. **Figure S3.** Establishment of artificial colonies under laboratory conditions. **Figure S4.** Effects of initial body weight of queens on fecundity for five days. **Figure S5.** Relationship between fresh body weight of queens and the proportion of polyploid cells in the fat body. **Figure S6.** Size distribution of sorted nuclei from the head and fat body of nymphoid queens.

## Data Availability

The raw data and materials are available upon request from the authors.
